# Avian eggshell biomineralization: an update on its structure, mineralogy and protein tool kit

**DOI:** 10.1186/s12860-021-00350-0

**Published:** 2021-02-12

**Authors:** J. Gautron, L. Stapane, N. Le Roy, Y. Nys, A. B. Rodriguez-Navarro, M. T. Hincke

**Affiliations:** 1 INRAE, Université de Tours, BOA, 37380 Nouzilly, France; 2 Departmento de Mineralogia y Petrologia, Universidad de Granada, 18071 Granada, Spain; 3 Department of Innovation in Medical Education, and Department of Cellular and Molecular Medicine, University of Ottawa, Ottawa, K1H8M5 Canada

**Keywords:** Chicken, Eggshell, Calcite, Biomineralization, Ion supply, Matrix protein functions, Amorphous calcium carbonate, Extracellular vesicles

## Abstract

**Background:**

The avian eggshell is a natural protective envelope that relies on the phenomenon of biomineralization for its formation. The shell is made of calcium carbonate in the form of calcite, which contains hundreds of proteins that interact with the mineral phase controlling its formation and structural organization, and thus determine the mechanical properties of the mature biomaterial. We describe its mineralogy, structure and the regulatory interactions that integrate the mineral and organic constituents during eggshell biomineralization.

**Main Body.**

We underline recent evidence for vesicular transfer of amorphous calcium carbonate (ACC), as a new pathway to ensure the active and continuous supply of the ions necessary for shell mineralization. Currently more than 900 proteins and thousands of upregulated transcripts have been identified during chicken eggshell formation. Bioinformatic predictions address their functionality during the biomineralization process. In addition, we describe matrix protein quantification to understand their role during the key spatially- and temporally- regulated events of shell mineralization. Finally, we propose an updated scheme with a global scenario encompassing the mechanisms of avian eggshell mineralization.

**Conclusion:**

With this large dataset at hand, it should now be possible to determine specific motifs, domains or proteins and peptide sequences that perform a critical function during avian eggshell biomineralization. The integration of this insight with genomic data (non-synonymous single nucleotide polymorphisms) and precise phenotyping (shell biomechanical parameters) on pure selected lines will lead to consistently better-quality eggshell characteristics for improved food safety. This information will also address the question of how the evolutionary-optimized chicken eggshell matrix proteins affect and regulate calcium carbonate mineralization as a good example of biomimetic and bio-inspired material design.

## Background

Oviparous avian species are characterized by extra-uterine development of the embryo in a closed chamber, the egg. The avian egg possesses a protective mineralized shell, which limits microbial contamination of its contents, and, thanks to its porosity, allows gaseous exchange between the external environment and the embryo. The distinctive features of an avian eggshell, as compared to bone or teeth, are the nature of the mineral deposit - calcium carbonate in the form of calcite, as well as the absence of cell-directed assembly during its fabrication. The avian eggshell is remarkable for its mechanical properties. In chickens, this thin mineral layer of about 0.3 mm thickness is capable of withstanding a static pressure of more than 3 kg; its formation during a 20 h period is one of the fastest known biomineralization processes. Our knowledge of eggshell mineralization has progressed significantly over the past 10 years, thanks to identification of the constituents of its organic matrix and the demonstration that they interact with calcium carbonate to determine its mineral phase crystallographic texture and therefore the mechanical properties of this biomaterial.

In this review, we describe recent knowledge on the structure and mineralogy of avian eggshells, with a focus on mechanisms and molecular actors that are involved in supplying the large quantity of ions necessary for its mineralization. Moreover, we provide an update on the identification and functional characterization of the proteins of the organic matrix that are involved in this biomineralization process.

## Main Text

### Eggshell structure and composition

#### Global structure of the avian eggshell

The shell has a highly ordered and mineralized structure and is rapidly formed (< 20 h for laying chickens) at physiological temperatures (< 40 °C) [1–4]. The thickness of the eggshell, the form and size of the whole eggshell and its structural elements, as well as features of the porous system vary among different species, but the general structure of the eggshell is basically the same in all birds [5, 6]. All avian eggshells are made of the trigonal phase of calcium carbonate, calcite, which is its most stable polymorph at room temperature. In the large majority of bird species, the mass of eggshell is proportional to the egg mass [7] and represents 10–11% of egg weight. The chicken eggshell has been the most studied to date. It contains 1.6% water, 3.3 to 3.5% organic matrix when eggshell membranes are included and 95% inorganic minerals. It is mainly made of calcium carbonate (98.4% of its mineral part), which is pervaded by an organic matrix corresponding to 2.3% of the shell weight. In addition to calcium (37.5%) and carbonate (58%), [2, 3], phosphorus is also present in the outer part and in the cuticle [8]. Moreover, numerous trace minerals (i.e. magnesium, manganese, copper, zinc) are found throughout the shell.

**Fig. 1:**
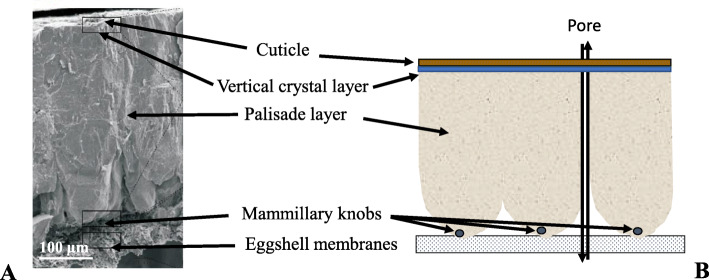
Chicken eggshell structure. **a** Scanning electronic microphotograph cross-fractured eggshell. **b** Corresponding labeled drawing of the different layers of the eggshell

The avian eggshell is composed of six layers as shown in Fig. 1 [1–4]. The innermost two layers are the uncalcified inner and outer shell membranes, which are composed of interlacing protein fibres. They support the mineralized shell and any disruption in eggshell membrane formation or structure prevents the normal mineralization of the eggshell [9]. The mineralized shell is anchored to nucleation sites, the mammillary knobs, which are organic rich structures distributed pseudoperiodically on surface of the outer eggshell membrane. Spherulitic aggregates of calcite crystals forms an array of inverted cones (mammillary layer) that fuse to form the compact palisade layer as mineralization proceeds. From this mammillary layer, the palisade layer, made of large columnar units (up to 200 μm in diameter) emerge from mammillary knobs. This palisade layer corresponding to two-thirds of the eggshell mineral thickness, ends at a thin vertical crystal layer perpendicular to the shell surface and located on the surface of the outer shell mineral [8]. The outermost layer, the cuticle, is a proteinaceous film deposited on the surface of the eggshell mineral. It contains hydroxyapatite crystals in its inner zone [8], as well as the bulk (2/3) of the superficial eggshell pigments [10]. Numerous pores penetrate the eggshell; their outer mouth is blocked by a cuticle plug in most species. The pores allow exchange of water and metabolic gases which is critical for embryonic development.

**Fig. 2:**
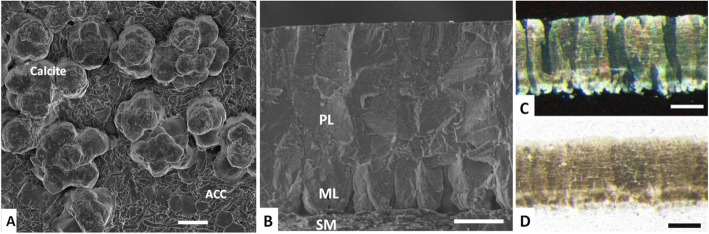
Eggshell ultrastructure and microstructure. **a** Scanning electron microscopy (SEM) image of an eggshell at an early stage of calcification (6 h post-ovulation.), showing aggregates of calcite crystals on mammillary knobs and ACC (amorphous calcium carbonate) flat-disk shaped particles on the shell membranes. **b** SEM image of cross-fractured eggshell showing the palisade layer (PL), mammillary layer (ML) and shell membranes (SM). Optical microscopy images of an eggshell cross-section: **c** as viewed under cross-polarized illumination, showing the columnar calcite crystal units of the mineral. **d** View under parallel light showing the distribution of the internal organic matter within the mineral. Scale bars are: **a** 10 μm; **b** 100 μm; **c** and **d** 200 μm

Eggshell biomineralization process follows a precise temporal and spatial control of its sequential formation. Upon ovulation, the yolk issues from the ovary and travels down the oviduct; it sequentially acquires the vitelline membrane in the infundibulum (15 min), the egg white in the magnum (3.5 h), the eggshell membranes in the white isthmus (1 h) and some organic aggregates (mammillary knobs) in the distal red isthmus before finally entering the uterus (19 h) [3, 10, 11]. The mammillary knobs deposited on the surface of the outer eggshell membranes are the sites of heterogeneous nucleation of calcium carbonate crystals. When it enters the uterus, the egg first acquires its final ovoid shape by hydration of the albumen (plumping), which elicits close contact of the outer eggshell membranes with the uterine mucosa. The avian eggshell mineralizes in this confined space, in an acellular uterine fluid that is supersaturated with respect to calcium and bicarbonate and contains the organic precursors of the shell matrix [2, 12, 13]. The concentrations of ionic and organic components vary during the sequential process of shell formation, i.e. the initiation of mineralization (5 h), linear deposition (12 h) and arrest of shell calcification (2 h) before egg expulsion [12]. The egg rotates during the linear deposition of calcium carbonate (0.33 g per hour in the chicken).

#### Avian eggshell ultrastructure and microstructure

The eggshell mineral composition is constant across all bird species, being always calcite (the most common calcium carbonate mineral). Eggshell mineralization begins with the deposition of massive mineral deposits of amorphous calcium carbonate (ACC) particles on the mammillary knobs (Fig. 1a); the ACC progressively dissolves and gives way to calcite crystals [14]. Although amorphous, ACC seems to have a short range proto-calcitic structure that predetermines its direct conversion into calcite. The formation of eggshell calcite crystals through the dissolution of an intermediate metastable amorphous mineral phase maintains a high supersaturation in the uterine fluid that sustains fast and continuous growth of calcite crystals during the linear phase of eggshell biomineralization. Although the uterine fluid is highly supersaturated with respect to all calcium carbonate polymorphs (aragonite, vaterite, calcite and ACC), only calcite is formed from metastable ACC, and ultimately is the sole mineral form observed in the mature eggshell, which indicates that there is a strict control over eggshell mineralogy [14, 15].

Figure 2b-d shows the ultra and microstructure characteristics of the eggshell as observed by scanning electron (SEM) and optical microscopy. The eggshell mineral part is about 320 μm thick (in chickens) and is made of dense columnar units (palisades) arranged perpendicular to the eggshell outer surface that arise from the cone shaped mammillary knobs that are attached to the shell membranes (Fig. 2b). Thin polished sections of eggshell, when viewed in cross-section under an optical microscopy, reveal palisades made of elongated calcite crystals, about 70–80 μm wide, that extend across the shell mineral thickness, and show varying degrees of light extinction due to differences in their crystallographic orientation (Fig. 2c). The eggshell possesses significant amounts of occluded organic matter (about 2%) that is distributed throughout the palisade region of the eggshell and is present at the highest concentration in the upper part of the mammillary layer, as can be observed under parallel light illumination (Fig. 2d)

**Fig. 3:**
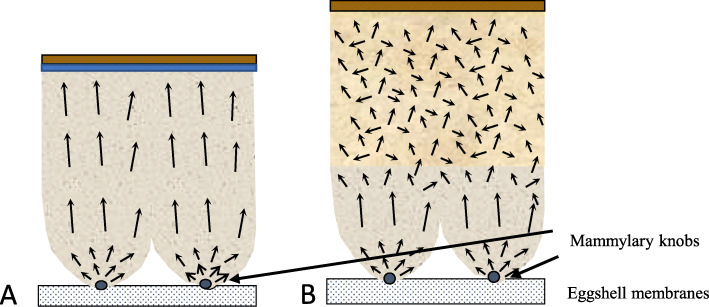
Comparison of schematic eggshell structure and crystal orientation in chicken (**a**) and in Guinea fowl (**b**) species. Black arrows represent the calcite crystal c-axes

The columnar crystal structure is generally observed for most bird eggshells, with small variation in the density and size of the columnar units. This columnar structure develops as calcite crystals from different nucleation sites (mammillary knobs), grow in size and impinge on each other, so they can only grow outward developing a columnar structure and a strong preferential orientation of calcite crystals, as in the case of ostrich eggshell [16, 17]. Guinea fowl is a notable exception. Its eggshell is about 500 μm thick and calcification follows the same pattern as for other birds, however, with a novel change in the size and orientation of crystals in the middle of the calcified layer [18, 19] (Fig. 3). In Guinea fowl eggshell, large columnar calcite units break into smaller crystal units with varying crystallographic orientation forming a microstructure with an intricate interlacing of calcite crystals (Fig. 3b). This particular structure is responsible for the exceptional mechanical properties of Guinea fowl eggshells by comparison to other birds.

### Molecular control of the avian eggshell biomineralization process

#### Regulation of mineral supply necessary for shell formation

**Fig. 4:**
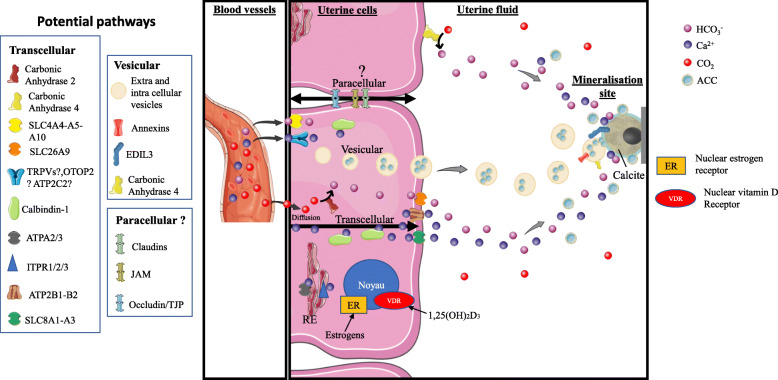
Comprehensive model for calcium and carbonate transport to the uterine fluid during eggshell calcification. The three potential pathways for ion transfer through uterine cells are transcellular, vesicular and paracellular mechanisms. They could function asynchronously or in an integrated fashion. The major protein players in each pathway are indicated on the figure (left). Graphical elements were from Servier Medical Art (https://smart.servier.com/), licensed under a Creative Commons Attribution 3.0 Unported License. Adapted from [20–25]

The calcium metabolism linked to egg formation in birds is strongly exaggerated. Indeed, there is no calcium storage in the shell gland (uterus) before shell formation [2, 3]. Calcium is directly provided by ionic blood calcium, to supply daily the necessary 2 g of shell calcium. This is a great metabolic challenge for an adult animal that weighs less than 2 kg. Calcium is provided by the hen diet, directly by intestinal absorption, although 40% of this is derived from bone mobilisation because of desynchronization between the period of feed intake (daytime) and shell formation, which mainly takes place during the night [3, 10]. This daily resorption of bone is facilitated in hens by the presence of a calcium reservoir, the medullary bone (about 12% of total bone calcium) (Fig. 4). The formation of medullary bone is induced in immature pullets by oestrogens and testosterone about 2 weeks before the onset of egg production [3, 20, 26]. During shell formation, medullary bone resorption is increased 9-fold, however osteoblastic activity reflecting medullary bone accretion is also activated (two-fold) to renew the medullary bone [27]. Supplying calcium for shell formation presents a challenge for calcium metabolism in the hen, which displays numerous physiological adaptations at sexual maturity. Birds develop a specific appetite for dietary calcium, with an accompanying increase in intestinal absorption of calcium. Medullary bone develops at the same time as the oviduct becomes sexually mature, with the capacity to secrete large amounts of calcium into the lumen of the distal segment (uterus). The second necessary ion forming the shell, the carbonate, originates from blood carbon dioxide, which is hydrated to bicarbonate ions by carbonic anhydrase (CA). Hens show respiratory hyperventilation during shell formation to alleviate metabolic acidosis due to acidification of uterine fluid and plasma during shell formation [28]. Both components of the shell mineral (Ca^2+^ and CO_3_^2−^) are continuously supplied during eggshell formation via the blood plasma, firstly by trans-epithelial ionic transport through the uterine epithelium [20, 29], and secondly, by vesicular secretion of ACC mineral particles [21].

#### Role of matrix proteins in the biomineralization process

The organic matrix of the eggshell plays a fundamental role in the manufacture of the shell and therefore in the establishment of its mechanical properties. The uterine fluid contains specialized biomolecules that specifically select calcite and stabilize ACC [14, 30]. In vitro experiments, in situ observations and genetic association analyses have confirmed the active control of eggshell mineralization by the components of the organic matrix.

A first experimental argument is the evidence for a change in the organic composition of the uterine fluid during the different phases of eggshell mineralization, which demonstrates that there is a modification in its protein inventory during the calcification process [12]. The organic matrix fraction contained in the shell has calcium-binding properties due to proteins [1, 31] or proteoglycans with keratan or dermatan sulfates [32]. Similarly, proteins in the uterine fluid have an affinity for calcium that can be demonstrated following electrophoresis [12]. The soluble organic fraction of the shell and of the uterine fluid modulates calcium carbonate precipitation in a dose-dependent manner [12, 32–34]. In vitro calcite crystallization assays demonstrate that uterine fluid increases the number of crystals produced, decreases their size, accelerates the mineralization kinetics and promotes the exclusive formation of calcite, which is the polymorphic type of calcium carbonate present in the mature eggshell [35, 36]. The morphology of calcite crystals is strongly modified by the presence of organic constituents extracted from the shell [34, 36] and by uterine fluid collected at the 3 main stages of shell biomineralization [12, 36].

Similar effects have been observed with single proteins isolated from the shell or uterine fluid. Calcite crystals are highly modified when grown in the presence of lysozyme, ovotransferrin, ovalbumin or ovocleidin-17 [37–41]. Goose eggshell ansocalcin is an ovocleidin-17 ortholog which is capable of inducing crystallization of calcite crystals in vitro [42, 43]. Ovoglycan, a dermatan sulfate proteoglycan with ovocleidin-116 as protein core, is polyanionic and acidic. It has a high affinity for calcium ions and therefore may modulate crystal growth during the formation of the palisade layer of the shell [39, 44].

Many physical parameters (hydrophobicity, charge...) modify the adsorption or repulsion mechanisms of these proteins on calcite crystal surfaces. Many eggshell proteins are preferentially absorbed on calcite crystal faces parallel to the c-axis and consequently alter the morphology of the growing calcite crystals to become elongated along the c-axis. These modifications affect the microstructure (size and orientation of the crystals) of the eggshell and therefore its mechanical properties. This hypothesis implies a variation in the quantity of specific proteins in the organic matrix that influence the mechanical properties of the shell. The existence of this relationship between organic matrix proteins and shell strength has been demonstrated in eggshell exhibiting various mechanical properties [45, 46].

There is also strong evidence that the structural organization of avian eggshell is under genetic control, as different avian species develop eggshells with specific microstructural characteristics [5, 17, 47, 48]. In fact, association studies between polymorphisms of genes encoding eggshell organic matrix proteins and eggshell phenotypes reveal that polymorphisms of specific shell proteins (ovalbumin, ovocleidin-116, ovocalyxin-32) partially explain variations in certain eggshell properties (i.e., eggshell thickness, crystal size, crystal orientation, eggshell mechanical properties) [49–51]. Quantitative Trait Loci (QTL) regions of the genome containing genes associated with shell morphological parameters have been identified on chromosomes 9 and Z in the chicken [52–54]. A recent study recorded a total of 118 QTLs associated with shell strength [51]. Among them, 24 are involved in fracture resistance [49, 54–58] and 33 in shell stiffness [49, 54, 59, 60]. In addition, a common QTL for the breaking strength characteristics and shell stiffness was found on chromosome 2 [54]. Other QTLs common to several shell quality parameters (shell weight, percentage of shell, shell thickness, fracture strength, and stiffness) have been identified [51]. Thus, the potential exists that eggshell quality can be improved by optimizing beneficial eggshell microstructure traits through genetic assisted selection programs. There is not yet a clear linkage between eggshell quality QTLs and genes encoding specific matrix proteins; however, progress is being made [61]. It is intriguing that eggs laid by aged hens exhibit modifications in eggshell ultrastructure and microstructure characteristics (i.e., type of defects, mammillary density; size of calcite crystal units) [62–64]. These structural changes are thought to be partially responsible for the marked decrease in eggshell mechanical properties in eggs from older hens. Interestingly, forced molting of older hens, which regenerates oviduct tissues, partially restores eggshell strength and reverses changes in shell structure [45].

#### Avian eggshell matrix proteins inventory

The eggshell membranes are synthesized and deposited in the isthmus segment of the oviduct, and provide a supportive substrate for the mineralizing eggshell. Eggshell membranes are composed of disulfide-rich protein fibers (~ 10% cysteine) that are coupled by irreversible lysine-derived crosslinks [65]. Collagen was revealed by identification of hydroxylysine and by immunochemistry [66]. However, the major shell membrane component is a Cysteine-Rich Eggshell Matrix Protein (abbreviated CREMP), which contains a large amount of cysteine [67, 68]. Collagen X, Lysyl oxidase-like 2 (LOXL2) and lysozyme are also present and the remaining (approximately 25%) is constituted by numerous proteins [69].

During eggshell mineralization, the proteins of the uterine fluid become progressively incorporated into the mineralizing eggshell [12]. These eggshell matrix proteins are a mixture of soluble and insoluble proteins, glyco- and phosphoproteins, and proteoglycans, which represent about 2% by weight of the calcified eggshell [70]. Eggshell matrix proteins were first explored using classical biochemistry methods, which identified 11 shell matrix proteins [70, 71]. Major advances came more recently, with the development of functional genomics-based methods that have allowed up to 900 specific eggshell proteins to be identified. The publication of the chicken (*Gallus gallus*) genome sequence [72] and corresponding mRNA sequences made possible investigations of egg compartment proteomes, using mass spectrometry-based high-throughput methods, as shown for the organic matrix of the chicken calcified eggshell layer [73].

The first major proteomic analysis of the chicken eggshell was published in 2006, generating a protein inventory for the acid-soluble eggshell organic matrix, followed by its phosphoproteome [74, 75], which together identified 528 different proteins as constituents of the soluble eggshell matrix. The insoluble fraction of the eggshell matrix was also investigated [76–79]. Rose-Martel et al. [80] performed a proteomic analysis of the outermost layer of the shell (cuticle), which is suspected to play a major role in preventing microbial penetration. More recent proteomic surveys allowed the identification of additional eggshell components [81], and the quantification of about 300 eggshell proteins at the key steps of shell calcification [30]. More recently, proteomic surveys have identified almost 500 eggshell membrane proteins [69, 82].

Proteomic studies have allowed the identification of hundreds of eggshell proteins. One drawback in this methodology is redundancy in protein annotation because proteomic studies have used identifiers originating from different databases. Altogether, chicken eggshell proteomic studies have described thousands of different protein identifiers, since one unique protein often possesses a number of identifiers. To address this problem, all protein sequences with different identifiers were aligned using a BLAST algorithm to eliminate all redundancies; with this approach, 904 single proteins were identified in the eggshell layers including membranes and cuticle [83]. Another integrated analysis of chicken eggshell matrix has enumerated a total of 69 phosphoproteins and 182 N-glycoproteins, for a total of 676 eggshell matrix proteins in the mineralized part [84].

Five additional bird eggshell proteomes have been studied quite extensively, identifying 697 proteins in turkey (*Meleagris gallopavo*) [85], 622 proteins in quail (*Coturnix japonica*) [86], 475 proteins in zebra finch (*Taeniopygia guttata*) [87], 484 proteins in duck (*Anas platyrhynchos*) and 149 proteins in Guinea fowl (*Numida meleagris*) [88]. Le Roy et al. [88] also compared these five bird eggshell proteomes and identified a common set of 119 proteins. However, the quality of the genomic databases and their annotation are a limiting factor in analysis of proteomic data for other bird species, since the chicken genome remains the best characterized of all birds.

### Overview of mineralization of avian eggshell

In the following parts, we have tried to integrate the different elements of current knowledge on both the regulation of organic and mineral inputs, as well as the role of major players in the organic matrix that allow the spatial-temporal regulation of shell deposition. The objective is to provide the reader with an updated and comprehensive model for mineralization of the avian eggshell. This scenario involves two distinct and complementary mechanisms that will be described in detail:

- The first element involves a large number of proteins necessary for an active and continuous supply of the ions necessary for shell mineralization within the acellular uterine fluid.
- The second aspect consists of temporally regulated secretion of proteins of the organic matrix, which regulate biomineralization of the shell.

These synchronized changes in uterine fluid concentrations of ionic and matrix protein precursors, with the organic constituents interacting with the developing mineral phase, result in the precise characteristics of the eggshell biomineral.

#### Active and continuous supply of the ions necessary for shell formation

Calcium secretion into the uterine lumen occurs against a concentration gradient mainly via the uterine glandular cells, as confirmed by the presence of calbindin [89] and carbonic anhydrase (CA) in these cells [90]. It also involves many transcellular transporters of other ionic species (Na^+^, K^+^, Cl^−^, H^+^), which participate in the process of calcium secretion and in the maintenance of cellular ionic homeostasis [3, 29, 91]. Changes in ion concentrations in uterine fluid throughout the stages of shell formation [15], in ion transfers in vitro and in vivo when ionic transport inhibitors are introduced [91], and early biochemical analyses exploring calbindin, carbonic anhydrase and Na^+^/K^+^-ATPase [89, 90], allow the main mechanisms of uterine ionic transfer to be defined. Transcriptomic approaches [22, 92] revealed a large number of uterine ionic transporters by analogies with protein sequences of transporters previously described in mammalian or avian tissues (intestine, kidney). This information was used to update the initial model of ionic transport for supplying mineral precursors of the shell to the uterine fluid [21–23]. Recently, an additional process to provide CaCO_3_ was revealed. Extracellular vesicles (EVs, 100–400 nm), originating from uterine epithelial cells, deliver stabilized amorphous calcium carbonate (ACC) to the mineralization front [21, 93].

We propose a comprehensive and further refined model for calcium and carbonate transport to the mineralization site during eggshell formation (Fig. 4). Calcium and carbon dioxide originate from the blood. Blood CO_2_ passively diffuses into uterine cells [24], where it is hydrated by CA2. Alternatively, bicarbonate can be actively transferred into uterine cells using the Na^+^/HCO_3_^−^ co-transporters SLC4A4-A5-A10 [20]. Bicarbonates are actively extruded from cells by the HCO_3_^−^/Cl^−^ exchanger SLC26A9 [20]. Additionally, bicarbonate ions can be directly produced in the uterine fluid by hydration of CO_2_ by membrane-bound CA4, which has its active site in the extracellular space [94]. The transcellular pathway to secrete calcium and bicarbonate ions into the fluid has been previously described [22, 23]. Plasma Ca^2+^ is transferred into uterine cells by transient receptor potential cation channels (TRPVs) and/or otopetrin 2 (OTOP2) and/or ATPase secretory pathway Ca^2+^ transporting 2 (ATP2C2) [20, 95]. Intracellular calcium ions are buffered/transferred by calbindin. Other Ca^2+^ pumps associated with the endoplasmic reticulum could also be involved in this transfer (ATP2A2/3 and ITPR1/2/3). Finally, the Ca^2+^/Na^2+^ exchangers SLC8A1–3 and the Ca^2+^ pumps ATP2B1-B2 are involved in the apical extrusion of calcium into the uterine fluid [20, 95]. Uterine Ca^2+^ secretion is quantitatively associated with calbindin levels and the regulation of uterine calcium transfer in conjunction with its synthesis has been studied in detail [20, 29]. The regulation of molecular actors involved in the supply of calcium in intestine, are dependent on the active metabolite of vitamin D [96]. However, this regulation is poorly documented for the avian uterus.

A paracellular Ca^2+^ uptake pathway is present in intestine [96] and acts to replenish calcium from dietary sources during eggshell biomineralization when soluble calcium in the intestinal lumen creates a favorable gradient for passive absorption This intestinal paracellular pathway involves claudins (CLDN), occludins (OCN), junctional adhesion molecules (JAM) and tight junction proteins (TJP) [96]. RNA-Seq analysis reveals the expression of several genes of this paracellular pathway (*Tjp1*, *Cldn1*, *Cldn10*, *Ocln*, *Jam2*) [97]. Moreover, expression of *Cldn10* has also been detected in chicken uterus [95, 98]. This paracellular pathway is probably contributing to the secretion of water and ions for osmotic regulation (K, Na) during the process of eggshell formation, and has been incorporated in our comprehensive model of ion transfer pathways during shell biomineralization (Fig. 4). The ionic calcium concentration in uterine fluid ranges from 6 to 10 mM depending of the stage of calcification [15], which is 6 times higher than blood calcium levels (1–2 mM); consequently, the concentration gradient is not in favor of calcium movement towards the uterine fluid through the paracellular pathway [20]. However, Bar [29] suggested that the electrical potential difference could invert this gradient, allowing some paracellular transfer of calcium into the uterine fluid [29].On the other hand, the concentration of potassium in uterine fluid (10 to 65 mM, dependent on the stage of mineralization) is higher than that of blood plasma (4 mM) [22]. Consequently, the paracellular pathway could participate in potassium transfer to maintain ionic and osmotic homeostasis.

**Fig. 5:**
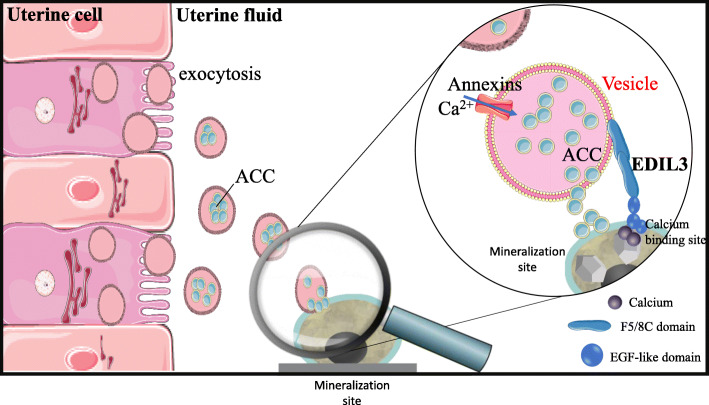
Proposed role for extracellular vesicles (EVs) in eggshell calcification. The EVs bud by exocytosis from the plasma membrane of the uterine cells. EVs transit the uterine fluid (UF) to deliver stabilized ACC (amorphous calcium carbonate) to the mineralization sites (MS). The passage of EV-encapsulated ACC avoids non-specific precipitation in the UF and provides stabilized ACC to the MS. Annexins allow calcium to penetrate into vesicles. EDIL3 (in bold) guides the EVs by targeting calcium to the mineralization front. Graphical elements were from Servier Medical Art (https://smart.servier.com/), licensed under a Creative Commons Attribution 3.0 Unported License. Adapted from Stapane [25]

Finally, we have added evidence related to vesicular ACC transport within the uterine fluid, and propose that ACC is taken up by vesicles that form inside uterine epithelial cells [21, 93]. Intracellular vesicles containing stabilized ACC are then secreted into the uterine fluid to be targeted to mineralization sites (Fig. 5). Numerous vesicular genes are highly expressed in the uterine tissue. Amongst annexin proteins, ANXA1 and ANXA2 were detected in cells of the epithelium, while ANXA8 and programmed cell death 6-interacting protein (PDCD6IP) were found in both tubular glands and epithelium. EGF-like repeat and discoidin I-like domain-containing protein 3 (EDIL3), CA4, syndecan-binding protein (SDCB) and numerous vesicular proteins were revealed by proteomic analysis of uterine fluid extracellular vesicles, which additionally were demonstrated to contain ACC (Stapane et al., 2019, 2020). In this model, we propose that annexins promote calcium entry into EVs, whereas CA4 catalyzes the hydration of CO_2_ into bicarbonate ions. ACC accumulates inside EVs and is delivered to the mineralization site, with EDIL3 and possibly MFGE8 as guidance molecules for this targeting [21, 93]. The quantitative contribution of the vesicular secretion of CaCO_3_ relative to the secretion of each ion remain to be explored. Additional calcium/bicarbonate ions mainly provided by the transcellular pathway would allow the growth of calcite at the mineralization front during the initial and subsequent stages of shell mineralization.

#### Temporal and spatial deposition of matrix proteins and calcite crystal orientation

**Fig. 6:**
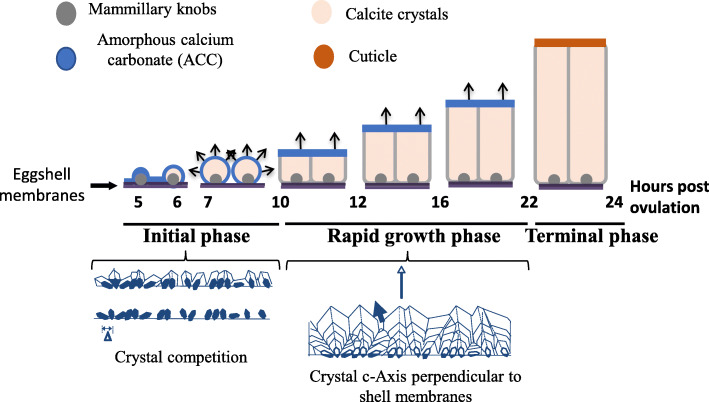
Schematic representation of the different stages of eggshell deposition. Mineralization starts by massive accumulation of ACC at 5 h post-ovulation. ACC is transformed into ACC aggregates (6 h p.o.), and larger crystal units then form with their c-axes progressively perpendicular to the surface (7–10 h p.o.). During the growth phase (10–22 h p.o.), the compact layer (palisade layer) is deposited with all crystals oriented perpendicular to the surface. Two hours before oviposition, arrest of mineralization occurs and the thin organic cuticle layer is deposited. Arrows indicate the orientation of the c-axis of calcite crystals

This process is well documented for biomineralization of the chicken eggshell and is extrapolated to illustrate the general features of avian eggshell formation (Fig. 6).

The first stage is the initiation phase (from 5 to 10 h after ovulation when the yolk enters the oviduct). When this phase begins, the eggshell weight is very low (0.18 g) compared to the final weight of the complete shell (about 5 g), and consists of the eggshell membrane fibers and their organic-rich rounded surface structures (mammillary knobs). This corresponds to the “slow” phase of mineralization, since 0.1 g of shell is deposited per hour and is divided into 3 events that are crucial and fundamental for the ultimate eggshell mechanical strength (Fig. 6). Initially, there is a calcification event (5 h post-ovulation), with the formation of flat disk-shaped ACC particles that nucleate over the entire membrane surface and in particular on the mammillary knobs, where they accumulate and form massive mineral deposits (Fig. 6). After this, ACC deposited at the organic nucleation sites gives way to aggregates of calcite microcrystals (6 h post-ovulation) (Fig. 6). At this point, calcite crystals are oriented in all directions (spherulites). During the next 3 h (7–10 h post-ovulation), calcite crystals grow to form the mammillary cone layer with the calcite crystal c-axis progressively oriented perpendicular to the surface. This occurs because the process of competitive growth between adjacent crystals favors the growth of vertically oriented crystals. Certain eggshell proteins inhibit the growth of crystal faces parallel to the c-axis so that calcite crystals became elongated along this direction. This anisotropy in crystal growth favors the emergence of crystals oriented with their c-axis (fastest growth direction) oriented roughly perpendicular to the egg surface, while crystals less favorably oriented become buried as they grow into adjacent crystals.

The second stage is the rapid-growth phase with a linear deposition of 0.33 g of calcite per hour. This phase is initiated when adjacent mammillary cones become fused (10 h post-ovulation), which forms a continuous compact layer (palisade layer) that continues to grow. This phase lasts 12 h, corresponding to deposition of about 4 g of eggshell which is organized as columnar calcite units with preferred orientation perpendicular to the eggshell surface. Experimental data (ionic speciation, crystal nanostructure) support the notion that ACC is continuously present at the shell mineralization front, explaining the extremely rapid growth kinetics of calcite crystal formation and eggshell deposition [14].

Finally, 2 h before oviposition, the terminal phase is characterized by the arrest of calcification and the deposition of a thin organic layer (cuticle), which constitutes a biofilm that covers the shell and obstructs the pore openings to restrict bacterial penetration through the shell.

**Fig. 7:**
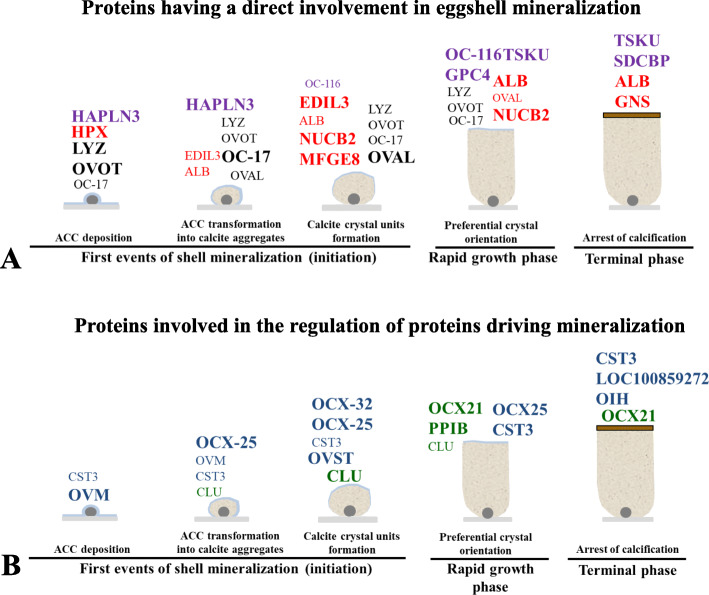
Schematic representation of the sequential events of mineralization and major matrix proteins at five time points during shell mineralization. The font size is relative to relative protein levels in the eggshell extract. **a** Proteins having a direct involvement in shell mineralization. Black lettering for proteins with established role in biomineralization. Red lettering for proteins with calcium-binding domains. Purple for proteoglycans and proteoglycan-binding proteins. **b** Proteins involved in the regulation of proteins directing mineralization. Green lettering for chaperone proteins and blue lettering for proteases and protease inhibitors. Adapted from Marie and coworkers [30, 99]

As reported above, organic matrix proteins have predominant roles in control of the different phases of shell calcification. Matrix proteins are involved in the stabilization of ACC, and to control the polymorphic mineral phase, morphology and size of crystals. Quantitative proteomics of the eggshell organic matrix coupled with bioinformatics analyses were used (i) to determine precisely the importance of particular proteins relative to these key events at the onset of biomineralization, and (ii) to predict the functional role of proteins in the stabilization of disordered forms of calcium carbonate and their influence on crystal polymorph and morphology [30, 99]. The spatial distribution and temporal variation in abundance of about 300 matrix proteins were correlated with the different phases of eggshell formation. Proteins having a direct involvement in shell mineralization (mineralizing proteins, able to bind calcium or divalent ions) were distinguished from proteins indirectly related to the calcification process (involved in the regulation of proteins directing mineralization) (Fig. 7).

#### Functions of key matrix protein candidates in avian eggshell mineralization

Among this large list of eggshell matrix proteins, the functions and roles ascribed to the biomineralization process have been proposed for a limited number of key protein candidates. As described below, some of these are particularly abundant in the eggshell and vary in abundance according to the different stages of eggshell formation [30] (Fig. 7).

##### Proteins having a direct involvement in shell mineralization

In this group are proteins with an established role in biomineralization. This group is made of proteins directly interacting with the mineral phase to stabilize ACC and/or to modify the morphology of crystals that determine the ultrastructure of avian eggshells.

The first category is made of three abundant egg white proteins with dual-roles in the egg (antimicrobial and mineralization activity). Indeed, lysozyme and ovotransferin are well known as antimicrobial proteins in the egg white, whereas several lines of evidence demonstrates that they are involved in the stabilization of ACC and influencing the morphology of calcite crystals during shell mineralization. Ovotransferrin (OVOT), lysozyme (LYZ) and ovalbumin (OVA) have repeatedly been inventoried as authentic eggshell matrix proteins during proteomic scans in chicken, quail, turkey, zebra finch and Guinea fowl eggshell [5, 38, 39, 78, 85, 86, 88, 99–101]. Gene association analysis revealed that OVA SNPs were associated with eggshell quality measurements of breaking strength and shell thickness [49]. OVA and OVOT are both genetically associated with variability in eggshell crystal size [50]. OVA modifies calcium carbonate precipitation in vitro and promotes unstable phases such as ACC [37, 102]. The role of OVA during eggshell formation may be to stabilize transient precursors of liquid ACC at the earliest stage of eggshell calcification and thereby prevent non-specific mineralization/precipitation [103]. The morphology of calcite crystals grown in vitro is dramatically altered in the presence of OVOT [38]. However, an antimicrobial protective role has often been ascribed to eggshell OVOT [80, 104]. LYZ is one of the major chicken eggshell matrix proteins. Calcite crystals grown in vitro in the presence of LYZ exhibited altered crystal morphology only at high concentrations [39]. Its role in the stabilization of ACC has been investigated but remains controversial. LYZ from eggshells of quail did not induce the precipitation of ACC under in vitro conditions [105]. However, metastable ACC particles were obtained in vitro in the presence of chicken egg white LYZ [102, 106]. LYZ considerably decreased the average diameter of metastable ACC particles and promoted a network of associated particles with incorporation of the protein into the precipitate [106]. In addition, LYZ-ACC particles reorganize exclusively into crystalline calcite [102, 106]. Conversely, LYZ was shown to be ineffective in the stabilization of ACC [103].

Ovocleidin-17 (OC-17) was the first chicken eggshell-specific matrix protein to be isolated, directly sequenced and to have its X-ray structure determined [41, 107–109]. OC-17 is an abundant matrix protein (40 μg/g of shell), which is concentrated in the inner mammillary cone layer where mineralization is initiated. It is a member of a family of homologous eggshell matrix proteins that have been identified in goose (ansocalcin), ostrich (struthiocalcin: SCA-1 & -2), emu (dromaiocalcin: DCA-1 & -2) and rhea (rheacalcin: RCA-1 &-2) [42, 110–112]. Moreover, Ovocleidin-17-like (OC-17-like) and dromaiocalcin-1-like (DCA-1-like) were recently identified in the Guinea fowl eggshell proteome [88]. These eggshell matrix proteins resemble C-type lectins and form a family of two related groups based on sequence identity, patterns of serine phosphorylation and conservation of cysteine residues. Although OC-17 protein was first identified in chicken, the chicken mRNA sequence was only recently determined by de novo transcriptomic assembly [113]. The OC-17 transcript has a high GC content (72.17%), which may explain the absence of its ortholog from genomic sequences in many avian species. Nevertheless, in zebra finch, two peptides were identified as partially homologous to chicken OC-17 [87]. In contrast to other eggshell matrix proteins implicated in mineralization (i.e. ovocleidin-116, osteopontin, EDIL3), OC-17 and its orthologs appear to be both avian- and oviduct/eggshell-specific. Temporal and spatial OC-17 mRNA expression analyses demonstrate that it is specifically expressed in the adult hen uterus during the laying cycle and barely at immature developmental stages [113]. The interaction of OC-17 with the calcite mineral phase has been evaluated in vitro and in silico [41, 114–116]. Calcite crystals grown in the presence of purified OC-17 display a modified crystalline habit [41]. Classical molecular dynamics simulations of OC-17 interaction with calcite predicts that specific residues interact with calcite stepped surfaces to control calcite nucleation; moreover, OC-17 appears to interact strongly with the surfaces of ACC and calcite nanoparticles and slabs via specific basic residues.

Osteopontin (OPN) is a phosphoglycoprotein member of the SIBLING family that is found in both bone and eggshell [1, 117]. OPN is a major actor in both hydroxyapatite and calcium carbonate mineralization in bone and shell, respectively. The oviduct expression of OPN is entirely uterine-specific and is temporally associated with eggshell calcification through coupling of physical distension of the uterus to osteopontin gene (Spp1) expression [118]. SNPs in Spp1 are associated with eggshell fracture toughness [49], and unusual patterns of uterine OPN expression are associated with eggshell mineralization defects [119]. Localization studies show that OPN is concentrated in the palisade layer of the eggshell, where it is associated with protein sheets of organic matrix [120–123]. Its association with parallel sheets of matrix, and more diffusely with the {104} crystallographic faces of eggshell calcite, is hypothesized to regulate crystal growth during mineralization. Specific OPN binding to the growing {104} rhombohedral calcite crystal face during mineralization could modify the resistance of the shell to fracture along this plane. The finding of an interaction between OPN and the {104} eggshell calcite faces was confirmed by in vitro studies of synthetic calcite growth, where inhibition by added OPN was observed at the {104} calcite crystal faces [121]. Nanoindentation and Atomic Force Microscopy (AFM) measurements suggest that OPN influences eggshell hardness and nanostructure, which in turn could control the mechanical properties of the shell [124].

Another important of category of eggshell proteins are the calcium-binding proteins (CaBPs) that interact with calcium to favor crystal nucleation and to specify the morphology of crystals. Bioinformatics investigations have associated numerous calcium-binding proteins to specific stages of shell calcification (proteins indicated in red on Fig. 7a). Amongst them are epidermal growth factor (EGF)-like repeats and discoidin-like domains 3 (EDIL3), for which a role in ACC vesicular transfer eggshell mineralization has recently been explored (Fig. 5) [21, 93]. In vertebrates, the *edil3* gene locus contains adjacent genes (*hapln1* and *vcan*) that are also involved in tissue calcification [93]. In chicken, the *edil3* gene is located on the chromosome Z [93] and EDIL3 protein was identified in 9 proteomic studies performed on eggshell or uterine fluid [30, 69, 74, 77, 78, 99, 125–127]. A recent in silico study emphasized the EGF-like and coagulation factor 5/8 (F5/8C) domains of EDIL3, which are proposed to bind calcium and extracellular vesicles during eggshell calcification [93]. Figure 5 incorporates EDIL3 as a guidance molecule to target EVs to the mineralization site during ACC vesicular transport. Milk fat globule EGF-factor 8 (MFGE8) and EDIL3 are paralogous proteins that share 69% similarity in amino acid sequence [93]. Although MFGE8 is also abundant in the hen eggshell, this protein seems to play a more ubiquitous functional role than EDIL3 [21]. Hemopexin (HPX), serum albumin (ALB), nucleobindin-2 (NUCB2) and gelsolin (GNS) are additional calcium-binding proteins that are overabundant in eggshell at key stages of shell calcification (Fig. 7a), but their potential role has not been investigated yet.

Proteoglycans are another category of major actors interacting directly with calcium during biomineralization that have been detected in eggshell [128–132]. These macromolecules combine a protein core with negatively charged complex polysaccharides, which strongly interact with calcium [133]. Hyaluronan and proteoglycan LiNk protein 3 (HAPLN3) [binds hyaluronic acid], glypican 4 (GPC4) [heparin sulfate proteoglycan], tsukushi (TSKU) [small leucine rich proteoglycan] and ovocleidin-116 (OC-116) [ovoglycan, dermatan sulfate proteoglycan] are abundant in chicken eggshell and have been directly correlated with specific shell mineralization events [30]. Functional roles have only been proposed for GPC4 and OC-116. Lavelin et al. [134] showed that *gpc4* expression in the chicken uterus only occurred during eggshell mineralization. OC-116 was first detected in the chicken uterine fluid, with high abundance, and then in the eggshell organic matrix [76, 135, 136]. This secreted protein is a dermatan sulfate proteoglycan, which also possess two N-glycosylated sites [137]. OC-116 is the most abundant chicken eggshell matrix protein, estimated at 80 μg/g of shell [86]. It is also present in embryonic chicken osteoblasts and osteoclasts, as well as young chick cortical bone, laying hen medullary bone and growth plate hypertrophic chondrocytes [138, 139]. This suggests an additional role for this protein in bone mineralization, a role which may be similar to that of its mammalian ortholog, matrix extracellular phosphoglycoprotein (MEPE). The OC-116/MEPE gene is a member of a vertebrate gene cluster, along with osteopontin (OPN/Spp1), bone sialoprotein (BSP) and dentin matrix protein 1 (DMP1), which together comprise the SIBLING (Small Integrin-Binding Ligand Interacting Glycoproteins) family that have a role in calcium biomineralization [140]. In mammals and non-avian reptiles, an additional gene encoding dentin sialophosphoprotein (DSPP) is involved in dentin formation. Its absence in avian genomes suggests its loss during evolution, which is coherent with the absence of teeth in birds [138]. Proteomics studies of avian eggshells have identified OC-116 as the most abundant matrix protein in chicken, turkey, quail and Guinea fowl [86, 88].

##### Proteins involved in the regulation of proteins directing mineralization

This group is made of uterine fluid proteins that interact with proteins directing mineralization. Indeed, mineralization takes place in an acellular medium and the proteins belonging to this group inhibit or activate proteins of the mineralization milieu. Some of these proteins may be involved in proper folding of eggshell matrix proteins to ensure an appropriate template for calcium and mineral interactions [141]. Many of these proteins have been described and quantified at the key stages of shell mineralization (Fig. 7b) [30, 99]. Nevertheless, there is still a lack of experimental evidence to demonstrate their specific role in the chicken eggshell mineralization process. Proteases and protease inhibitors are believed to play specific and controlled roles during the calcification process, either by degrading proteins or regulating processing of proteins into their mature forms. Ovocalyxin-32 (OCX-32) belongs to this group, and was first identified in the chicken eggshell [142]. Its gene (RARRES1) is highly expressed in the uterus and the isthmus region of the oviduct. In the eggshell, OCX-32 is abundant in the outer palisade layer, the vertical crystal layer and the cuticle, and it is present in the insoluble fraction of the eggshell organic matrix [79]. OCX-32 is most abundant in chicken uterine fluid during the initial phase of mineralization [30]. OCX-32 possesses 32% identity with the mammalian carboxypeptidase inhibitor, latexin, and the retinoic acid receptor-responder 1 RARRES1/TIG1 [142, 143]. Recombinant OCX-32 inhibits bovine carboxypeptidase activity and slows the growth of *Bacillus subtilis* [144], implying an antimicrobial role that would provide protection for the developing avian embryo in the fertilized egg. OCX-32 is very abundant in the non-mineralized cuticle of the shell, playing a major role in the antimicrobial properties of the cuticle [80]. SNP studies of the chicken OCX-32 gene revealed its association with eggshell quality traits [49, 145, 146]. With respect to other avian species, OCX-32 protein has not been identified in quail and turkey eggshell [85, 86]; however, it is found in the organic matrix of zebra finch, Guinea fowl and duck eggshell [88, 101, 147].

Ovocalyxin-36 (OCX-36) is a prominent 36 kDa protein present in the uterine fluid collected during the active calcification stage of chicken eggshell mineralization [148]. OCX-36 message is expressed in the oviduct segments where eggshell formation takes place (isthmus and uterus), and is strongly upregulated during eggshell calcification [148]. OCX-36 localizes to the calcified eggshell and its cuticle, and is abundant in the shell membranes [30, 69, 79, 80, 125, 148]. The OCX-36 protein sequence displays significant identity with mammalian proteins such as lipopolysaccharide-binding proteins (LBP), bactericidal permeability-increasing proteins (BPI) and palate, lung and nasal epithelium clone (Plunc) family proteins that are key components of the innate immune system and act as a first line of host defense [148, 149]; however, the OCX-36 gene member of this family is specific to birds [143]. LBP proteins initiate the inflammatory host response upon the detection of a pathogen. OCX-36 may therefore participate in natural defense mechanisms that keep the egg and oviduct free of pathogens. This hypothesis is supported by observations that the purified protein binds bacterial lipopolysaccharide (LPS) and lipoteichoic acid (LTA), and inhibits *S. aureus* bacterial growth [150]. Purified OCX-36 and OCX-36 derived peptides differentially modulate innate immune responses in vitro (macrophage cell culture) and in vivo (mouse model of endotoxemia) [151]. OCX-36 is one of the most abundant constituents of the chicken, turkey, quail and zebra finch eggshell proteomes [85, 86, 101]. However, the impact of the purified protein upon calcite crystallization has not yet been evaluated.

## Conclusion

The avian eggshell is a composite material with a calcitic mineral phase and an organic phase whose interactions determine its ultrastructure and resulting mechanical properties. The egg is a widely consumed food throughout the world with more than 1300 billion units produced per year. It is therefore a basic food and an important agricultural product; whose shell quality is critically important for human food security. For this reason, the mechanism of shell formation has been intensely studied. The functional information reviewed in this article must now be associated with genomic data. Several collaborative programs are currently in progress with poultry breeding industrialists that integrate recent advances in our knowledge of the mechanisms of eggshell mineralization with advances in genomic selection to bring precision to the selection of animals capable of laying eggshells with enhanced mechanical properties under a variety of physiological and production conditions. Because of its interest to agriculture and human food production, the eggshell represents the best understood calcium carbonate biomineralization model. It is therefore also an excellent model for the development of new bioinspired materials. The functional motifs identified in proteins that regulate eggshell mineralization will be excellent additives for synthesis of novel structures with tunable material properties.

## Data Availability

The datasets generated in our labs are available. The microarray data were deposited in the NCBI Gene Expression Omnibus (GEO) data repository under accession number GSE 52823 and GSE17267. The mass spectrometry proteomic data have been deposited into the ProteomeXchange Consortium via the PRIDE partner repository with the dataset identifiers PXD001450 and PXD000992.

## Abbreviations

ACC: Amorphous calcium carbonate
SEM: Scanning electron microscopy
CA: Carbonic anhydrase
QTLs: Quantitative Trait Loci
SNPs: Single Nucleotide Polymorphisms
CREMP: Cysteine-Rich Eggshell Matrix Protein
LOXL2: Collagen X, Lysyl oxidase-like 2
TRPVs: Transient receptor potential cation channels
OTOP2: Otopetrin 2
ATP2C2: ATPase secretory pathway Ca^2+^ transporting 2
CLDN: Claudins
OCN: Occludins
JAM: Junctional adhesion molecules
TJP: Tight junction proteins (
ANXA: Annexin
PDCD6IP: Programmed cell death 6-interacting protein
EDIL3: EGF-like repeat and discoidin I-like domain-containing protein 3
SDCB: Syndecan-binding protein
OVOT: Ovotransferrin
LYZ: Lysozyme
OVA: Ovalbumin
OC-17: Ovocleidin-17
OPN/SPP1: Osteopontin
AFM: Atomic Force Microscopy
CaBPs: Calcium-binding proteins
EGF: Epidermal growth factor
MFGE8: Milk fat globule EGF-factor 8
HPX: Hemopexin
ALB: Serum albumin
NUCB2: Nucleobindin-2
GNS: Gelsolin
HAPLN3: Hyaluronan and proteoglycan LiNk protein 3
GPC4: Glypican 4
TSKU: Tsukushi
OC-116: Ovocleidin-116
MEPE: Matrix extracellular phosphoglycoprotein
BSP: Bone sialoprotein
DMP1: Dentin matrix protein 1
SIBLING: Small Integrin-Binding Ligand Interacting Glycoproteins
DSPP: Dentin sialophosphoprotein
OCX-32: Ovocalyxin-32
OCX-36: Ovocalyxin-36
LBP: Lipopolysaccharide-binding proteins
BPI: Bactericidal permeability-increasing proteins
Plunc: Palate, lung and nasal epithelium clone
LPS: Bacterial lipopolysaccharide
LTA: Lipoteichoic acid
EVs: Extracellular vesicles
PL: Palisade layer
ML: Mammillary layer
SM: Shell membranes
UF: Uterine fluid
MS: Mineralization sites

## References

[1] Hincke MT, Nys Y, Gautron J, Mann K, Rodriguez-Navarro AB, McKee MD (2012). The eggshell: structure, composition and mineralization. Front Biosci.

[2] Nys Y, Hincke MT, Arias JL, Garcia-Ruiz JM, Solomon SE (1999). Avian eggshell mineralization. Poult Avian Biol Rev.

[3] Sauveur B, Derevier M (1988). Reproduction des volailles et production d'oeufs Quae edn.

[4] Solomon SE (1991). Egg and egg quality.

[5] Panheleux M, Bain M, Fernandez MS, Morales I, Gautron J, Arias JL, Solomon SE, Hincke M, Nys Y (1999). Organic matrix composition and ultrastructure of eggshell: a comparative study. Brit Poultry Sci.

[6] Romanoff L, Romanoff J (1949). The avian egg. New york (USA): John Winley and sons, INc.

[7] Ar A, Rahn H, Paganelli VC (1979). The avian egg: mass and strength. Condor.

[8] Dennis JE, Xiao SQ, Agarwal M, Fink DJ, Heuer AH, Caplan AI (1996). Microstructure of matrix and mineral components of eggshells from white leghorn chickens (Gallus gallus). J Morphol.

[9] Chowdurry SD (1990). Shell membrane system in relation to lathyrogen toxicity and copper deficiency. Wourld Poult Sci J.

[10] Nys Y, Guyot N, Nys Y, Bain M, Vanimmerseel F (2011). Egg formation and chemistry. Improving the safety and quality of eggs and egg products.

[11] Gilbert AB, King A, Mc Lelland J (1979). Form and function in birds. Female genital organs.

[12] Gautron J, Hincke MT, Nys Y (1997). Precursor matrix proteins in the uterine fluid change with stages of eggshell formation in hens. Connect Tissue Res.

[13] Nys Y, Gautron J, Garcia-Ruiz JM, Hincke MT (2004). Avian eggshell mineralization: biochemical and functional characterization of matrix proteins. Cr Palevol.

[14] Rodriguez-Navarro AB, Marie P, Nys Y, Hincke MT, Gautron J (2015). Amorphous calcium carbonate controls avian eggshell mineralization: a new paradigm for understanding rapid eggshell calcification. J Struct Biol.

[15] Nys Y, Zawadzki J, Gautron J, Mills AD (1991). Whitening of brown-shelled eggs: mineral composition of uterine fluid and rate of protoporphyrin deposition. Poult Sci.

[16] Rodriguez-Navarro A, Garcia-Ruiz JM (2000). Model of textural development of layered crystal aggregates. Eur J Mineral.

[17] Rodriguez-Navarro AB, Yebra A, Nys Y, Jimenez-Lopez C, Garcia-Ruiz JM (2007). Analysis of avian eggshell microstructure using X-ray area detectors. Eur J Mineral.

[18] Panheleux M, Kalin O, Gautron J, Nys Y (1999). Features of eggshell formation in Guinea fowl: kinetics of shell deposition, uterine protein secretion and uterine histology. Br Poult Sci.

[19] Perez-Huerta A, Dauphin Y (2016). Comparison of the structure, crystallography and composition of eggshells of the Guinea fowl and graylag goose. Zoology.

[20] Nys Y, Le Roy N, Vitamin D, Feldman D (2018). Calcium homeostasis and eggshell biomineralization in female chicken.

[21] Stapane L, Le Roy N, Ezagal J, Rodriguez-Navarro AB, Labas V, Combes-Soia L, Hincke MT, Gautron J. Avian eggshell formation reveals a new paradigm for vertebrate mineralization via vesicular amorphous calcium carbonate. J Biol Chem. 2020, In press:doi. 10.1074/jbc.RA1120.014542. 10.1074/jbc.RA120.014542 PMC7681030 32816992

[22] Jonchere V, Brionne A, Gautron J, Nys Y (2012). Identification of uterine ion transporters for mineralisation precursors of the avian eggshell. BMC Physiol.

[23] Brionne A, Nys Y, Hennequet-Antier C, Gautron J. Hen uterine gene expression profiling during eggshell formation reveals putative proteins involved in the supply of minerals or in the shell mineralization process. BMC Genomics. 2014;15:220. 10.1186/1471-2164-15-220 PMC3999959 24649854

[24] Hodges RD, Lörcher K (1967). Possible Sources of the Carbonate Fraction of Egg Shell Calcium Carbonate. Nature.

[25] Stapane L (2019). Biominéralisation de la coquille d’œuf de poule : mise en évidence d’un transport vésiculaire du minéral impliquant les protéines EGF-like repeats and discoidinlike domains 3 (EDIL3) et Milk fat globule EGF-factor 8 (MFGE8).

[26] Dake CG, Sugiyama T, Gay C. The Role of Hormones in the Regulation of Bone Turnover and Eggshell Calcification. In: Scanes CG, editor. Sturkie's avian physiology 6Th edition. Vol. 40, issue 2. Elsevier academic place; 2015. p. 576–603.

[27] Van de Velde JP, Vermeiden JP, Touw JJA, Veldhuijzen JP (1984). Changes in activity of chicken medullary bone cell populations in relation to the egg-laying cycle. Metab Bone Dis Relat Res.

[28] Mongin P, Piiper JE (1978). Acid-base balance during eggshell formation. Respiratory, function of birds, adult and embryonic.

[29] Bar A (2009). Calcium transport in strongly calcifying laying birds: mechanisms and regulation. Comp Biochem Physiol A Mol Integr Physiol.

[30] Marie P, Labas V, Brionne A, Harichaux G, Hennequet-Antier C, Rodriguez-Navarro AB, Nys Y, Gautron J (2015). Quantitative proteomics provides new insights into chicken eggshell matrix protein functions during the primary events of mineralisation and the active calcification phase. J Proteome.

[31] Abatangelo G, Dagagordini D, Castellani I, Cortivo R (1978). Some observations on the calcium-ion binding to the eggshell matrix. Calc Tiss Res.

[32] Arias JL, Carrino DA, Fernandez MS, Rodriguez JP, Dennis JE, Caplan AI (1992). Partial biochemical and immunochemical characterization of avian eggshell extracellular matrices. Arch Biochem Biophys.

[33] Arias JL, Fink DJ, Xiao SQ, Heuer AH, Caplan AI (1993). Biomineralization and eggshells: cell-mediated acellular compartments of mineralized extracellular matrix. Int Rev Cytol.

[34] Gautron J, Bain M, Solomon S, Nys Y (1996). Soluble matrix of hen's eggshell extracts changes in vitro the rate of calcium carbonate precipitation and crystal morphology. Br Poult Sci.

[35] Dominguez-Vera JM, Gautron J, Garcia-Ruiz JM, Nys Y (2000). The effect of avian uterine fluid on the growth behavior of calcite crystals. Poult Sci.

[36] Hernandez-Hernandez A, Gomez-Morales J, Rodriguez-Navarro AB, Gautron J, Nys Y, Garcia-Ruiz JM (2008). Identification of some active proteins in the process of hen eggshell formation. Cryst Growth Des.

[37] Dombre C, Guyot N, Moreau T, Monget P, Da Silva M, Gautron J, Rehault-Godbert S (2017). Egg serpins: the chicken and/or the egg dilemma. Semin Cell Dev Biol.

[38] Gautron J, Hincke MT, Panheleux M, Garcia-Ruiz JM, Boldicke T, Nys Y (2001). Ovotransferrin is a matrix protein of the hen eggshell membranes and basal calcified layer. Connect Tissue Res.

[39] Hincke MT, Gautron J, Panheleux M, Garcia-Ruiz J, McKee MD, Nys Y (2000). Identification and localization of lysozyme as a component of eggshell membranes and eggshell matrix. Matrix Biol.

[40] Jimenez-Lopez C, Rodriguez-Navarro A, Dominguez-Vera JM, Garcia-Ruiz JM (2003). Influence of lysozyme on the precipitation of calcium carbonate: a kinetic and morphologic study. Geochim Cosmochim Ac.

[41] Reyes-Grajeda JP, Moreno A, Romero A (2004). Crystal structure of ovocleidin-17, a major protein of the calcified Gallus gallus eggshell: implications in the calcite mineral growth pattern. J Biol Chem.

[42] Lakshminarayanan R, Kini RM, Valiyaveettil S (2002). Investigation of the role of ansocalcin in the biomineralization in goose eggshell matrix. Proc Natl Acad Sci U S A.

[43] Lakshminarayanan R, Valiyaveettil S, Rao VS, Kini RM (2003). Purification, characterization, and in vitro mineralization studies of a novel goose eggshell matrix protein, ansocalcin. J Biol Chem.

[44] Fernandez MS, Moya A, Lopez L, Arias JL (2001). Secretion pattern, ultrastructural localization and function of extracellular matrix molecules involved in eggshell formation. Matrix Biol.

[45] Ahmed AMH, Rodriguez-Navarro AB, Vidal ML, Gautron J, Garcia-Ruiz JM, Nys Y (2005). Changes in eggshell mechanical properties, crystallographic texture and in matrix proteins induced by moult in hens. Brit Poultry Sci.

[46] Panheleux M, Nys Y, Williams J, Gautron J, Boldicke T, Hincke MT (2000). Extraction and quantification by ELISA of eggshell organic matrix proteins (ovocleidin-17, ovalbumin, ovotransferrin) in shell from young and old hens. Poult Sci.

[47] Dalbeck P, Cusack M (2006). Crystallography (electron backscatter diffraction) and chemistry (electron probe microanalysis) of the avian eggshell. Cryst Growth Des.

[48] Silyn-Roberts H, Sharp RM (1986). Crystal-growth and the role of the organic network in eggshell biomineralization. Proc R Soc Ser B-Bio.

[49] Dunn IC, Joseph NT, Bain M, Edmond A, Wilson PW, Milona P, Nys Y, Gautron J, Schmutz M, Preisinger R (2009). Polymorphisms in eggshell organic matrix genes are associated with eggshell quality measurements in pedigree Rhode Island red hens. Anim Genet.

[50] Dunn IC, Rodriguez-Navarro AB, Mcdade K, Schmutz M, Preisinger R, Waddington D, Wilson PW, Bain MM (2012). Genetic variation in eggshell crystal size and orientation is large and these traits are correlated with shell thickness and are associated with eggshell matrix protein markers. Anim Genet.

[51] Rome H, Le Roy P (2016). Chromosomal regions influencing egg production and egg quality traits in hens. Inra Productions Animales.

[52] Ankra-Badu G, Aggrey S (2005). Identification of candidate genes at quantitative trait loci on chicken chromosome Z using orthologous comparison of chicken, mouse, and human genomes. Poult Sci.

[53] Takahashi H, Yang D, Sasaki O, Furukawa T, Nirasawa K (2009). Mapping of quantitative trait loci affecting eggshell quality on chromosome 9 in an F-2 intercross between two chicken lines divergently selected for eggshell strength. Anim Genet.

[54] Tuiskula-Haavisto M, Honkatukia M, Preisinger R, Schmutz M, de Koning DJ, Wei WH, Vilkki J (2011). Quantitative trait loci affecting eggshell traits in an F-2 population. Anim Genet.

[55] Jiang RS, Xie Z, Chen XY, Geng ZY (2010). A single nucleotide polymorphism in the parathyroid hormone gene and effects on eggshell quality in chickens. Poult Sci.

[56] Liu W, Li D, Liu J, Chen S, Qu L, Zheng J, Xu G, Yang N. A genome-wide SNP scan reveals novel loci for egg production and quality traits in white leghorn and brown-egg dwarf layers. PLoS One. 2011;6(12):e28600. 10.1371/journal.pone.0028600 PMC3234275 22174844

[57] Sasaki O, Odawara S, Takahashi H, Nirasawa K, Oyamada Y, Yamamoto R, Ishii K, Nagamine Y, Takeda H, Kobayashi E (2004). Genetic mapping of quantitative trait loci affecting body weight, egg character and egg production in F2 intercross chickens. Anim Genet.

[58] Yao JF, Chen ZX, Xu GY, Wang XL, Ning ZH, Zheng JX, Qu LJ, Yang N (2010). Low-density lipoprotein receptor-related protein 8 gene association with egg traits in dwarf chickens. Poult Sci.

[59] Takahashi H, Sasaki O, Nirasawa K, Furukawa T (2010). Association between ovocalyxin-32 gene haplotypes and eggshell quality traits in an F-2 intercross between two chicken lines divergently selected for eggshell strength. Anim Genet.

[60] Wolc A, Arango J, Jankowski T, Dunn I, Settar P, Fulton JE, O'Sullivan NP, Preisinger R, Fernando RL, Garrick DJ (2014). Genome-wide association study for egg production and quality in layer chickens. J Anim Breed Genet.

[61] Zhang F, Yin ZT, Zhang JF, Zhu F, Hincke M, Yang N, Hou ZC (2020). Integrating transcriptome, proteome and QTL data to discover functionally important genes for duck eggshell and albumen formation. Genomics.

[62] Park JA, Sohn SH (2018). The influence of hen aging on eggshell ultrastructure and Shell mineral components. Korean J Food Sci Anim Resour.

[63] Roberts JR, Chousalkar K (2013). Samiullah: egg quality and age of laying hens: implications for product safety. Anim Prod Sci.

[64] Rodriguez-Navarro A, Kalin O, Nys Y, Garcia-Ruiz JM (2002). Influence of the microstructure on the shell strength of eggs laid by hens of different ages. Br Poult Sci.

[65] Leach J, Roland M (1982). Biochemistry of the organic matrix of the eggshell. Poult Sci.

[66] Arias JL, Fernandez MS, Dennis JE, Caplan AI (1991). Collagens of the chicken eggshell membranes. Connect Tissue Res.

[67] Du JW, Hincke MT, Rose-Martel M, Hennequet-Antier C, Brionne A, Cogburn LA, Nys Y, Gautron J (2015). Identifying specific proteins involved in eggshell membrane formation using gene expression analysis and bioinformatics. Bmc Genomics.

[68] Kodali VK, Gannon SA, Paramasivam S, Raje S, Polenova T, Thorpe C. A Novel Disulfide-Rich Protein Motif from Avian Eggshell Membranes. PLoS One. 2011;6(3):e18187. 10.1371/journal.pone.0018187 PMC3068167 21479176

[69] Ahmed TA, Suso HP, Hincke MT (2017). In-depth comparative analysis of the chicken eggshell membrane proteome. J Proteome.

[70] Hincke MT, Bernard AM, Lee ER, Tsang CP, Narbaitz R (1992). Soluble protein constituents of the domestic fowl's eggshell. Br Poult Sci.

[71] Gautron J, Nau F, Mann K, Guerin-Dubiard C, Rehault S, Hincke MT, Nys Y (2007). Molecular approaches for the identification of novel egg components. World Poultry Sci J.

[72] Hillier LW, Miller W, Birney E, Warren W, Hardison RC, Ponting CP, Bork P, Burt DW, Groenen MAM, Delany ME (2004). Sequence and comparative analysis of the chicken genome provide unique perspectives on vertebrate evolution. Nature.

[73] Gautron J, Réhault-Godbert S, Nys Y, Nys Y, Bain M, Van Immerseel F (2011). Use of High-throughput technology to identify new egg components. Improving the safety and quality of eggs and egg products.

[74] Mann K, Macek B, Olsen JV (2006). Proteomic analysis of the acid-soluble organic matrix of the chicken calcified eggshell layer. Proteomics.

[75] Mann K, Olsen JV, Macek B, Gnad F, Mann M (2007). Phosphoproteins of the chicken eggshell calcified layer. Proteomics.

[76] Miksik I, Eckhardt A, Sedlakova P, Mikulikova K (2007). Proteins of insoluble matrix of avian (Gallus Gallus) eggshell. Connect Tissue Res.

[77] Mikšík I, Ergang P, Pácha J (2014). Proteomic analysis of chicken eggshell cuticle membrane layer. Anal Bioanal Chem.

[78] Miksik I, Sedlakova P, Lacinova K, Pataridis S, Eckhardt A (2010). Determination of insoluble avian eggshell matrix proteins. Anal Bioanal Chem.

[79] Mikšík I, Sedláková P, Mikulíková K, Eckhardt A, Kašička V (2007). Comparison of CE-MS and LC-MS analyses of avian eggshell matrix proteins. Chromatographia.

[80] Rose-Martel M, Du J, Hincke MT (2012). Proteomic analysis provides new insight into the chicken eggshell cuticle. J Proteome.

[81] Sun C, Xu G, Yang N. Differential label-free quantitative proteomic analysis of avian eggshell matrix and uterine fluid proteins associated with eggshell mechanical property. Proteomics. 2013;13:3523-36. 10.1002/pmic.201300286 24151251

[82] Makkar S, Liyanage R, Kannan L, Packialakshmi B, Lay JO, Rath NC (2015). Chicken egg Shell membrane associated proteins and peptides. J Agric Food Chem.

[83] Gautron J. Proteomics Analysis of Avian Eggshell Matrix Proteins: Toward New Advances on Biomineralization. Proteomics. 2019;19(13):220. 10.1002/pmic.201900120 31125177

[84] Yang R, Geng F, Huang X, Qiu N, Li SG, Teng H, Chen L, Song HB, Huang Q. Integrated proteomic, phosphoproteomic and N-glycoproteomic analyses of chicken eggshell matrix. Food Chem. 2020;330:127167. 10.1016/j.foodchem.2020.127167 32531632

[85] Mann K, Mann M. The proteome of the calcified layer organic matrix of Turkey (Meleagris gallopavo) eggshell. Proteome Sci. 2013;11:40. 10.1186/1477-5956-11-40 PMC3766105 23981693

[86] Mann K, Mann M. Proteomic analysis of quail calcified eggshell matrix: a comparison to chicken and Turkey eggshell proteomes. Proteome Sci. 2015;13:29. 10.1186/s12953-015-0078-1 PMC4550075 26312056

[87] Mann K (2015). The calcified eggshell matrix proteome of a songbird, the zebra finch (Taeniopygia guttata). Proteome Sci.

[88] Le Roy N, Combes-Soia L, Brionne A, Labas V, Rodriguez-Navarro A, Hincke M, Nys Y, Gautron J. Guinea fowl eggshell quantitative proteomics yield new findings related to its unique structural characteristics and superior mechanical properties. J Proteome. 2019. 10.1016/j.jprot.2019.103511 31493547

[89] Wasserman RH, Smith CA, Smith CM, Brindak ME, Fullmer CS, Krook L, Penniston JT, Kumar R (1991). Immunohistochemical localization of a calcium pump and calbindin-D28k in the oviduct of the laying hen. Histochemistry.

[90] Bernstein RS, Nevalainen T, Schraer R, Schraer H (1968). Intracellular distribution and role of carbonic anhydrase in the avian (Gallus domesticus) shell gland mucosa. Biochim Biophys Acta.

[91] Eastin WC, Spaziani E (1978). On the mechanism of calcium secretion in the avian shell gland (uterus). Biol Reprod.

[92] Jonchere V, Rehault-Godbert S, Hennequet-Antier C, Cabau C, Sibut V, Cogburn LA, Nys Y, Gautron J (2010). Gene expression profiling to identify eggshell proteins involved in physical defense of the chicken egg. BMC Genomics.

[93] Stapane L, Le Roy N, Hincke MT, Gautron J (2019). The glycoproteins EDIL3 and MFGE8 regulate vesicle-mediated eggshell calcification in a new model for avian biomineralization. J Biol Chem.

[94] Zhu XL, Sly WS (1990). Carbonic anhydrase IV from human lung. Purification, characterization, and comparison with membrane carbonic anhydrase from human kidney. J Biol Chem.

[95] Sah N, Kuehu DL, Khadka VS, Deng Y, Peplowska K, Jha R, Mishra B (2018). RNA sequencing-based analysis of the laying hen uterus revealed the novel genes and biological pathways involved in the eggshell biomineralization. Sci Rep.

[96] Gloux A, Le Roy N, Brionne A, Bonin E, Juanchich A, Benzoni G, Piketty ML, Prie D, Nys Y, Gautron J (2019). Candidate genes of the transcellular and paracellular calcium absorption pathways in the small intestine of laying hens. Poult Sci.

[97] Gautron J, Stapane L, Rodriguez-Navarro A, Nys Y, Hincke MT (2020). New insights on eggshell mineralization and how they can contribute to maintain shell quality. Poultry Science Association, 2020 PSA annual meeting: July 20-22, 2020*;* Virtual conference.

[98] Yin Z, Lian L, Zhu F, Zhang ZH, Hincke M, Yang N, Hou ZC. The transcriptome landscapes of ovary and three oviduct segments during chicken (Gallus gallus) egg formation. Genomics. 2019. 10.1016/j.ygeno.2019.02.003 30772430

[99] Marie P, Labas V, Brionne A, Harichaux G, Hennequet-Antier C, Nys Y, Gautron J (2015). Quantitative proteomics and bioinformatic analysis provide new insight into protein function during avian eggshell biomineralization. J Proteome.

[100] Hincke MT. Ovalbumin is a component of the chicken eggshell matrix. Connect Tissue Res. 1995;31:227-33. 10.3109/03008209509010814 15609630

[101] Mann K (2015). The calcified eggshell matrix proteome of a songbird, the zebra finch (Taeniopygia guttata). Proteome Sci.

[102] Wang XQ, Wu CM, Tao K, Zhao K, Wang JQ, Xu H, Xia DH, Shan HH, Lu JR (2010). Influence of ovalbumin on CaCO3 precipitation during in vitro biomineralization. J Phys Chem B.

[103] Wolf SE, Leiterer J, Pipich V, Barrea R, Emmerling F, Tremel W. Strong stabilization of amorphous calcium carbonate emulsion by ovalbumin: gaining insight into the mechanism of polymer-induced liquid precursor processes. J Am Chem Soc. 2011;133:12642–9. 10.1021/ja202622g PMC3170880 21736300

[104] Wellman-Labadie O, Picman J, Hincke MT (2008). Antimicrobial activity of cuticle and outer eggshell protein extracts from three species of domestic birds. Brit Poultry Sci.

[105] Lakshminarayanan R, Loh XJ, Gayathri S, Sindhu S, Banerjee Y, Kini RM, Valiyaveettil S (2006). Formation of transient amorphous calcium carbonate precursor in quail eggshell mineralization: an in vitro study. Biomacromolecules.

[106] Voinescu AE, Touraud D, Lecker A, Pfitzner A, Kunz W, Ninham BW (2007). Mineralization of CaCO3 in the presence of egg white lysozyme. Langmuir.

[107] Hincke MT, Tsang CP, Courtney M, Hill V, Narbaitz R (1995). Purification and immunochemistry of a soluble matrix protein of the chicken eggshell (ovocleidin 17). Calcif Tissue Int.

[108] Mann K, Siedler F (1999). The amino acid sequence of ovocleidin 17, a major protein of the avian eggshell calcified layer. Biochem Mol Biol Int.

[109] Reyes-Grajeda JP, Jauregui-Zuniga D, Rodriguez-Romero A, Hernandez-Santoyo A, Bolanos-Garcia VM, Moreno A (2002). Crystallization and preliminary X-ray analysis of ovocleidin-17 a major protein of the Gallus gallus eggshell calcified layer. Protein and Peptide Letters.

[110] Mann K (2004). Identification of the major proteins of the organic matrix of emu (Dromaius novaehollandiae) and rhea (Rhea americana) eggshell calcified layer. Brit Poultry Sci.

[111] Mann K, Siedler F (2004). Ostrich (*Struthio camelus*) eggshell contains two different C-type lectin-like proteins. Isolation, amino acid sequence, and post-translational modifications. Biochim Biophys Acta.

[112] Mann K, Siedler F (2006). Amino acid sequences and phosphorylation sites of emu and rhea eggshell C-type lectin-like proteins. Comp Biochem Physiol B: Biochem Mol Biol.

[113] Zhang Q, Liu L, Zhu F, Ning Z, Hincke MT, Yang N. Integrating de novo transcriptome assembly and cloning to obtain chicken ovocleidin-17 full-length cDNA. PLoS One. 2014;9:e93452. 10.1371/journal.pone.0093452 PMC3968166 24676480

[114] Freeman CL, Harding JH, Quigley D, Rodger PM. Simulations of ovocleidin-17 binding to calcite surfaces and its implications for eggshell formation. J Phys Chem. 2011;115.

[115] Freeman CL, Harding JH, Quigley D, Rodger PM (2012). Protein binding on stepped calcite surfaces: simulations of ovocleidin-17 on calcite {31.16} and {31.8}. Phys Chem Chem Phys.

[116] Freeman CL, Harding JH, Quigley D, Rodger PM. How does an amorphous surface influence molecular binding? Ovocleidin-17 and amorphous calcium carbonate. Phys Chem Chem Phys. 2015;17:7287-95. 10.1039/c5cp00434a 26009013

[117] Sodek J, Ganss B, McKee MD (2000). Osteopontin. Crit Rev Oral Biol Med.

[118] Lavelin I, Yarden N, Ben-Bassat S, Bar A, Pines M (1998). Regulation of osteopontin gene expression during egg shell formation in the laying hen by mechanical strain. Matrix Biol.

[119] Arazi H, Yoselewitz I, Malka Y, Kelner Y, Genin O, Pines M (2009). Osteopontin and calbindin gene expression in the eggshell gland as related to eggshell abnormalities. Poult Sci.

[120] Chien YC, Hincke MT, McKee MD (2009). Avian eggshell structure and osteopontin. Cells Tissues Organs.

[121] Chien YC, Hincke MT, Vali H, McKee MD (2008). Ultrastructural matrix-mineral relationships in avian eggshell, and effects of osteopontin on calcite growth in vitro. J Struct Biol.

[122] Fernandez MS, Escobar C, Lavelin I, Pines M, Arias JL (2003). Localization of osteopontin in oviduct tissue and eggshell during different stages of the avian egg laying cycle. J Struct Biol.

[123] Hincke MT, Chien YC, Gerstenfeld LC, McKee MD (2008). Colloidal-gold immunocytochemical localization of osteopontin in avian eggshell gland and eggshell. J Histochemistry Cytochemistry.

[124] Athanasiadou D, Jiang W, Goldbaum D, Saleem A, Basu K, Pacella MS, Bohm CF, Chromik RR, Hincke MT, Rodriguez-Navarro AB (2018). Nanostructure, osteopontin, and mechanical properties of calcitic avian eggshell. Sci Adv.

[125] Cordeiro CMM, Hincke MT (2016). Quantitative proteomics analysis of eggshell membrane proteins during chick embryonic development. J Proteome.

[126] Kaweewong K, Garnjanagoonchorn W, Jirapakkul W, Roytrakul S (2013). Solubilization and identification of hen eggshell membrane proteins during different times of chicken embryo development using the proteomic approach. Protein J.

[127] Rose-Martel M, Smiley S, Hincke MT (2015). Novel identification of matrix proteins involved in calcitic biomineralization. J Proteome.

[128] Carrino DA, Dennis JE, Wu TM, Arias JL, Fernandez MS, Rodriguez JP, Fink DJ, Heuer AH, Caplan AI (1996). The avian eggshell extracellular matrix as a model for biomineralization. Connect Tissue Res.

[129] Carrino DA, Rodriguez JP, Caplan AI (1997). Dermatan sulfate proteoglycans from the mineralized matrix of the avian eggshell. Connect Tissue Res.

[130] Fernandez MS, Araya M, Arias JL (1997). Eggshells are shaped by a precise spatio-temporal arrangement of sequentially deposited macromolecules. Matrix Biol.

[131] Nakano T, Ikawa N, Ozimek L (2001). Extraction of glycosaminoglycans from chicken eggshell. Poult Sci.

[132] Nakano T, Ikawa N, Ozimek L (2002). Galactosaminoglycan composition in chicken eggshell. Poult Sci.

[133] Hunter GK, Wong KS, Kim JJ (1988). Binding of calcium to glycosaminoglycans: an equilibrium dialysis study. Arch Biochem Biophys.

[134] Lavelin I, Meiri N, Einat M, Genina O, Pines M (2002). Mechanical strain regulation of the chicken glypican-4 gene expression in the avian eggshell gland. Am J Physiol Regul Integr Comp Physiol.

[135] Hincke MT, Gautron J, Tsang CPW, McKee MD, Nys Y (1999). Molecular cloning and ultrastructural localization of the core protein of an eggshell matrix proteoglycan, ovocleidin-116. J Biol Chem.

[136] Mann K, Hincke MT, Nys Y (2002). Isolation of ovocleidin-116 from chicken eggshells, correction of its amino acid sequence and identification of disulfide bonds and glycosylated Asn. Matrix Biol.

[137] Nimtz M, Conradt HS, Mann K (2004). LacdiNAc (GalNAcbeta1-4GlcNAc) is a major motif in N-glycan structures of the chicken eggshell protein ovocleidin-116. Biochim Biophys Acta.

[138] Bardet C, Vincent C, Lajarille M-C, Jaffredo T, Sire J-Y. OC-116, the chicken ortholog of mammalian MEPE found in eggshell, is also expressed in bone cells. J Exp Zool. 2010;314B:653-62. 10.1002/jez.b.21366 20665709

[139] Horvat-Gordon M, Yu F, Burns D, Leach RM. Ovocleidin (OC-116) is present in avian skeletal tissues. Poult Sci. 2008;87. 10.3382/ps.2008-00031 18648057

[140] Rowe PSN. The chicken or the egg: PHEX, FGF23 and SIBLINGs unscrambled. Cell Biochem Funct. 2012;30:355-75. 10.1002/cbf.2841 PMC3389266 22573484

[141] Mann K, Gautron J, Nys Y, McKee MD, Bajari T, Schneider WJ, Hincke MT (2003). Disulfide-linked heterodimeric clusterin is a component of the chicken eggshell matrix and egg white. Matrix Biol.

[142] Gautron J, Hincke MT, Mann K, Panhéleux M, Bain M, McKee MD, Solomon SE, Nys Y (2001). Ovocalyxin-32, a novel chicken eggshell matrix protein: isolation, amino acid sequencing, cloning and immunocytochemical localization. J Biol Chem.

[143] Tian X, Gautron J, Monget P, Pascal G (2010). What makes an egg unique? Clue from evolutionary scenarios of egg-specific genes. Biol Reprod.

[144] Xing J, Wellman-Labadie O, Gautron J, Hincke MT (2007). Recombinant eggshell ovocalyxin-32: expression, purification and biological activity of the glutathione S-transferase fusion protein. Comp Biochem Physiol B: Biochem Mol Biol.

[145] Fulton JE, Soller M, Lund AR, Arango J, Lipkin E (2012). Variation in the ovocalyxin-32 gene in commercial egg-laying chickens and its relationship with egg production and egg quality traits. Anim Genet.

[146] Uemoto Y, Suzuki C, Sato S, Sato S, Ohtake T, Sasaki O, Takahashi H, Kobayashi E (2009). Polymorphism of the ovocalyxin-32 gene and its association with egg production traits in the chicken. Poult Sci.

[147] Zhu F, Zhang F, Hincke M, Yin Z-T, Chen S-R, Yang N, Hou Z-C (2019). iTRAQ-Based Quantitative Proteomic Analysis of Duck Eggshell During Biomineralization. Proteomics.

[148] Gautron J, Murayama E, Vignal A, Morisson M, McKee MD, Rehault S, Labas V, Belghazi M, Vidal ML, Nys Y (2007). Cloning of ovocalyxin-36, a novel chicken eggshell protein related to lipopolysaccharide-binding proteins, bactericidal permeability-increasing proteins, and plunc family proteins. J Biol Chem.

[149] Gautron J, Rehault-Godbert S, Pascal G, Nys Y, Hincke MT (2011). Ovocalyxin-36 and other LBP/BPI/PLUNC-like proteins as molecular actors of the mechanisms of the avian egg natural defences. Biochem Soc Trans.

[150] Cordeiro CMM, Esmaili H, Ansah G, Hincke MT. Ovocalyxin-36 is a pattern recognition protein in chicken eggshell membranes. PLoS One. 2013;8:e84112. 10.1371/journal.pone.0084112 PMC3877205 24391897

[151] Kovacs-Nolan J, Cordeiro C, Young D, Mine Y, Hincke MT. Ovocalyxin-36 is an effector protein modulating the production of proinflammatory mediators. Vet Immunol Immunopathol. 2014;160:1-11. 10.1016/j.vetimm.2014.03.005 24803310

