# Correction to: Avian eggshell biomineralization: an update on its structure, mineralogy and protein tool kit

**DOI:** 10.1186/s12860-021-00351-z

**Published:** 2021-02-22

**Authors:** J. Gautron, L. Stapane, N. Le Roy, Y. Nys, A. B. Rodriguez-Navarro, M. T. Hincke

**Affiliations:** 1 INRAE, Université de Tours, BOA, 37380 Nouzilly, France; 2 Departmento de Mineralogia y Petrologia, Universidad de Granada, 18071 Granada, Spain; 3 Department of Innovation in Medical Education, and Department of Cellular and Molecular Medicine, University of Ottawa, Ottawa, K1H8M5 Canada

**BMC Mol Cell Biol 22, 11 (2021)**

**https://doi.org/10.1186/s12860-021-00350-0**

Following publication of the original article [1], an error in the structure of the abstract was reported. The updated Abstract is as follows:

The avian eggshell is a natural protective envelope that relies on the phenomenon of biomineralization for its formation. The shell is made of calcium carbonate in the form of calcite, which contains hundreds of proteins that interact with the mineral phase controlling its formation and structural organization, and thus determine the mechanical properties of the mature biomaterial. We describe its mineralogy, structure and the regulatory interactions that integrate the mineral and organic constituents. We underline recent evidence for vesicular transfer of amorphous calcium carbonate (ACC), as a new pathway to ensure the active and continuous supply of the ions necessary for shell mineralization. Currently more than 900 proteins and thousands of upregulated transcripts have been identified during chicken eggshell formation. Bioinformatic predictions address their functionality during the biomineralization process. In addition, we describe matrix protein quantification to understand their role during the key spatially- and temporally- regulated events of shell mineralization. Finally, we propose an updated scheme with a global scenario encompassing the mechanisms of avian eggshell mineralization. With this large dataset at hand, it should now be possible to determine specific motifs, domains or proteins and peptide sequences that perform a critical function during avian eggshell biomineralization. The integration of this insight with genomic data (non-synonymous single nucleotide polymorphisms) and precise phenotyping (shell biomechanical parameters) on pure selected lines will lead to consistently better-quality eggshell characteristics for improved food safety. This information will also address the question of how the evolutionary-optimized chicken eggshell matrix proteins affect and regulate calcium carbonate mineralization as a good example of biomimetic and bio-inspired material design.

The original article [1] has been corrected.
